# Glucose-6-Phosphate Dehydrogenase Deficiency Activates Endothelial Cell and Leukocyte Adhesion Mediated via the TGFβ/NADPH Oxidases/ROS Signaling Pathway

**DOI:** 10.3390/ijms21207474

**Published:** 2020-10-10

**Authors:** Rajesh Parsanathan, Sushil K. Jain

**Affiliations:** Department of Pediatrics and Center for Cardiovascular Diseases and Sciences, Louisiana State University Health Sciences Center-Shreveport, 1501 Kings Highway, Shreveport, LA 71130, USA; sjain@lsuhsc.edu

**Keywords:** endothelial dysfunction, glucose-6-phosphate dehydrogenase deficiency, oxidative stress, transforming growth factor-beta, NADPH oxidase, reactive oxygen species, leukocyte adhesion, ICAM-1, VCAM-1

## Abstract

Glucose-6-phosphate dehydrogenase (G6PD) deficiency, the most common genetic inherited trait among humans, affects ~7% of the global population, and is associated with excess risk of cardiovascular disease (CVD). Transforming growth factor-β (TGF-β) regulates immune function, proliferation, epithelial-mesenchymal transition, fibrosis, cancer, and vascular dysfunction. This study examined whether G6PD deficiencies can alter TGF-β-mediated NADPH oxidases (NOX) and cell adhesion molecules (CAM) in human aortic endothelial cells (HAEC). Results show that treatment with high glucose and the saturated free fatty acid palmitate significantly downregulated G6PD; in contrast, mRNA levels of TGF-β components, NOX and its activity, and reactive oxygen species (ROS) were significantly upregulated in HAEC. The expression levels of TGF-β and its receptors, NOX and its activity, and ROS were significantly higher in HG-exposed G6PD-deficient cells (G6PD siRNA) compared to G6PD-normal cells. The protein levels of adhesion molecules (ICAM-1 and VCAM-1) and inflammatory cytokines (MCP-1 and TNF) were significantly increased in HG-exposed G6PD-deficient cells compared to G6PD-normal cells. The adherence of monocytes (SC cells) to HAEC was significantly elevated in HG-treated G6PD-deficient cells compared to control cells. Pharmacological inhibition of G6PD enhances ROS, NOX and its activity, and endothelial monocyte adhesion; these effects were impeded by NOX inhibitors. The inhibition of TGF-β prevents NOX2 and NOX4 mRNA expression and activity, ROS, and adhesion of monocytes to HAEC. L-Cysteine ethyl ester (cell-permeable) suppresses the mRNA levels of TGF-β and its receptors, along with NOX2 and NOX4, and decreases NOX activity, ROS, and adhesion of monocytes to HAEC. This suggests that G6PD deficiency promotes TGF-β/NADPH oxidases/ROS signaling, the expression of ICAM-1 and VCAM-1, and the adhesion of leukocytes to the endothelial monolayer, which can contribute to a higher risk for CVD.

## 1. Introduction

Glucose-6-phosphate dehydrogenase (G6PD) deficiency is the most common diverse X-linked human enzymopathy genetic trait [1]. G6PD deficiency, which is caused by roughly 160 missense amino acids, affects an estimated 400 million people worldwide. Males are either G6PD deficient or normal, but females exhibit a broader range of G6PD deficiencies, from severe deficiency to normal [2]. Recent studies among U.S. military personnel and civilians have shown that G6PD deficiency is associated with an increased incidence of cardiovascular disease (CVD) [3,4]. The prevalence and association between G6PD deficiency and the risk of CVD in the Mediterranean region were validated in a large cohort (9604) of a propensity score-matched study carried out in a large population from Northern Sardinia [5]. Another cross-sectional study in a Chinese population showed that 154917 G6PD-deficient females aged 20–49 had a higher risk for elevated blood pressure and hypertension during pre-pregnancy and pregnancy. They also showed that G6PD deficiency was more obviously associated with elevated systolic blood pressure [6].

The incidence of G6PD deficiency among African Americans (AA) is 11–14%, compared with only 1% in Caucasians. The AA population is also known to have the highest incidence of CVD and 2–3 times the risk of stroke compared with other ethnic groups [7]. The G6PD A^−^ variant associated with mild to a moderate enzyme deficiency (class III) with residual enzyme activity (10–60%) is present/recognized predominantly in AA [4]. The high prevalence of hypertension and diabetes among Afro Caribbeans in the West may be directly related to the incidence of G6PD deficiency in these populations [8], and it is more severe in AA, as they tend to develop these conditions earlier in life. G6PD-deficient AA subjects are known to have elevated levels of oxidative stress (OS) and lower levels of glutathione (GSH) [9]. Studies of G6PD deficiency have shown that altering redox homeostasis plays a role in the predisposition of the system to defective vasodilation and the progression of cardiovascular disease [8,10,11]. Endothelial activation and monocyte adhesion and infiltration contribute significantly to pro-atherogenic responses, vascular inflammation (a forerunner of vascular dysfunction), and CVD [12].

Reactive oxygen species (ROS) activated profibrotic transforming growth factor-β (TGF-β) regulates immune function, proliferation, and epithelial-mesenchymal transition (EndMT) and is implicated in the initiation and progression of fibrosis, cancer, and vascular dysfunction [13,14]. However, no previous study has examined the biochemical and molecular mechanisms that would demonstrate whether TGF-β signaling leads to the endothelial activation/dysfunction in G6PD-deficient status.

The foremost aim of this study was to evaluate the effect of G6PD deficiency (to mimic G6PD A^−^ variant; mild-moderate) on TGF-β/NADPH oxidases/ROS signaling and cell adhesion molecules (CAM) in human aortic endothelial cells (HAEC) and monocyte-endothelial adhesion. This study enlarges our understanding of how G6PD deficiency impairs vascular redox homeostasis, which may lead to novel therapeutic strategies to reduce vascular dysfunction and thereby the incidence of CVD in vulnerable populations.

## 2. Results

### 2.1. High Glucose and Palmitate Mitigate G6PD and Increases Oxidative Stress

Relative to the control and mannitol groups, high glucose and palmitate (metabolic insult) treated HAEC groups displayed impaired G6PD expression and activity (Figure S1A–C). This reduced activity of G6PD, which may contribute to increased oxidative stress, reduces glutathione content, upregulates mRNA levels of TGF-β components, and escalates NOX2/4 genes activity (Figure S1A,D–F). Further metabolic insults facilitate increased monocyte-endothelial cell adhesion (Figure S1G,H). These results suggest that metabolic insults mediated G6PD deficiency leading to activation of the endothelium via the ROS/TGF-β/NOX system (Figure S1I).

### 2.2. G6PD Deficiency Exacerbates the Effects of High Glucose

**Figure 1:**
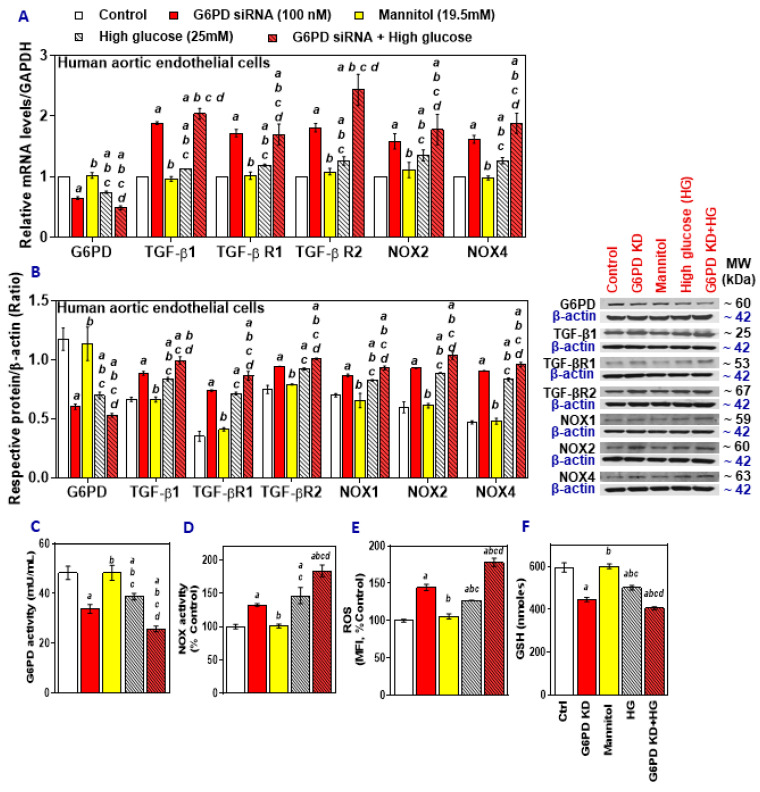
The effect of treatment with high glucose on Glucose-6-phosphate dehydrogenase (G6PD), TGF-β1, TGF-β1 receptors, and NADPH oxidases (NOX) in G6PD-normal and G6PD-deficient human aortic endothelial cells (HAEC). (**A**) RT-qPCR was performed to assess the level of target genes as indicated. (**B**) Representative Western blot analysis (G6PD, TGF-β1, TGF-βR1, TGF-βR2, NOX2, and NOX4) performed on total protein. The left panel represents the semi-quantitative analysis of the ratio of protein abundance to β-actin. (**C**) G6PD activity. (**D**) NADPH oxidase activity. (**E**) reactive oxygen species (ROS) and (**F**) glutathione (GSH) content. Results are mean ± SEM (*n* = 3–6). Significance at *p* < 0.05: a, compared with control; b, compared with G6PD siRNA; c, compared with mannitol; d, compared with high glucose.

G6PD knockdown and treatment with HG both decreased the expression of G6PD mRNA in HAEC, and the combination exacerbated these effects, while TGF-β1, TGF-β R1, TGF-β R2, NOX2, and NOX4 mRNA were upregulated (Figure 1A). These effects were also seen in protein expression; G6PD was downregulated, and TGF-β1, TGF-β R1, TGF-β R2, NOX2, and NOX4 showed increased expression in the G6PD siRNA group, HG group, and the combination group (Figure 1B). G6PD activity also decreased in the G6PD siRNA treated cells, as well as the HG and HG with G6PD siRNA treated groups (Figure 1C), while the NOX activity (Figure 1D) and ROS levels increased (Figure 1E), and the GSH content decreased (Figure 1F) in these groups. Knockdown of G6PD and treatment with high glucose mimicked the phenotype of G6PD deficiency and diabetes, with more detrimental effects in combination compared to either condition alone. 

### 2.3. G6PD Deficiency and Treatment with High Glucose Increases Cell Adhesion Molecules and Inflammatory Cytokines in HAEC In Vitro

**Figure 2:**
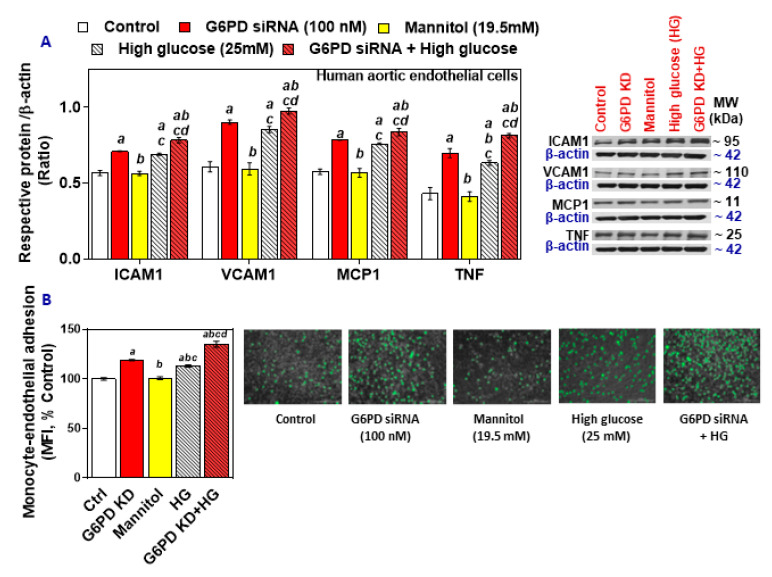
The effect of treatment with high glucose on cell adhesion molecules (ICAM-1, VCAM-1), MCP-1, and TNF in G6PD-normal and G6PD-deficient HAEC and monocyte-endothelial cell adhesion. (**A**) Representative Western blot analysis (ICAM-1, VCAM-1, MCP-1, and TNF) performed on total protein. The left panel represents the semi-quantitative analysis of the ratio of protein abundance to β-actin. (**B**) Phase-contrast images of HAEC and SC monocytic cells (scale bar: 200 μm). Results are mean ± SEM (*n* = 3–6). Significance at *p* < 0.05: a, compared with control; b, compared with G6PD siRNA; c, compared with mannitol; d, compared with high glucose.

Protein levels of ICAM-1, VCAM-1, MCP-1, and TNF were upregulated in the G6PD-deficient HAEC, as well as in the HG group, and the effect was much higher in the G6PD-deficient group exposed to high glucose (Figure 2A). Monocyte-endothelial cell adhesion was also increased in the G6PD-deficient cells and the HG-treated cells (Figure 2B). Thus, the G6PD deficiency, along with hyperglycemia, may favor endothelial dysfunction by increasing levels of inflammatory cytokines, upregulating cell adhesion molecules, and facilitating the adhesion of monocytes.

### 2.4. TGF-β Inhibitors Lower NOX2/4 Expression and Activity and Oxidative Stress in G6PD-Deficient HAEC 

**Figure 3:**
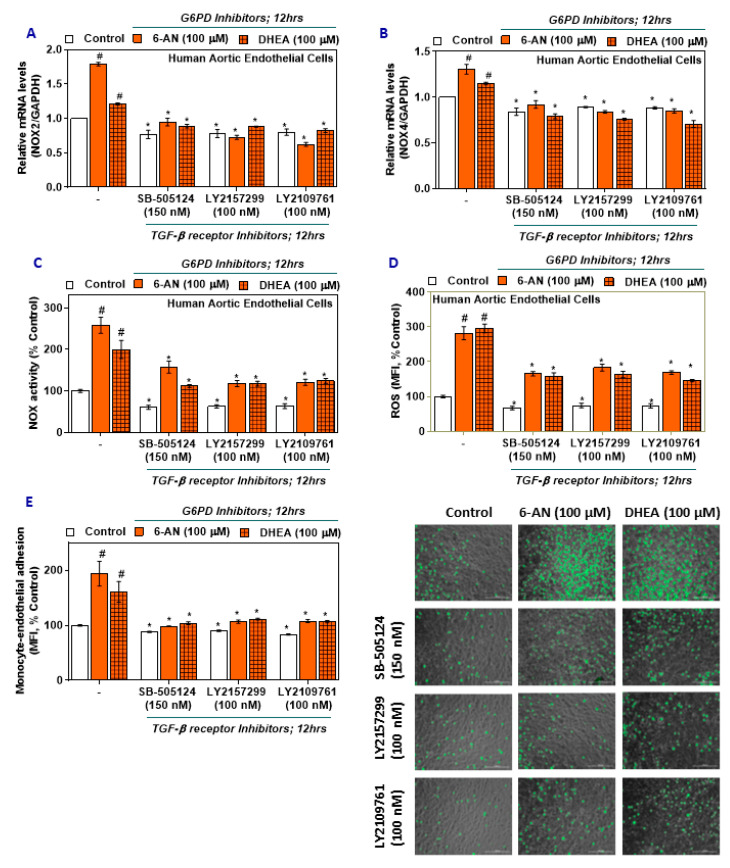
The effect of G6PD and TGF-β receptor pharmacological inhibitors on NOX2 and NOX4 expression, NADPH oxidase activity, ROS in HAEC, and monocyte-endothelial cell adhesion. (**A**,**B**) NOX2 and NOX4 mRNA. (**C**) NADPH oxidase activity. (**D**) ROS. (**E**) Phase-contrast images of HAEC and SC monocytic cells (scale bar: 200 μm). Results are mean ± SEM (*n* = 3–6). Significance at *p* < 0.05: #, compared with control–G6PD inhibitor alone groups; *, compared with control–TGF-β receptor inhibitor groups.

G6PD-deficient HAEC treated with TGF-β inhibitors (SB-505124, LY2157299, and LY2109761) lowered the expression of NOX2 mRNA (Figure 3A) and NOX4 mRNA (Figure 3B) across all groups, with the G6PD-deficient HAEC still upregulated compared to the G6PD-normal HAEC. NOX activity was decreased in G6PD-deficient TGF-β inhibitor-treated groups (Figure 3C), and ROS was also decreased in the G6PD-deficient TGF-β inhibitor-treated groups (Figure 3D). Interestingly, these observations indicate that inhibition of TGF-β signaling impedes NOX2 and 4 expression and thereby decreases oxidative stress in HAEC.

### 2.5. TGF-β Inhibitors Reduce Monocyte-Endothelial Adhesion in G6PD-Deficient HAEC

To further confirm the impact of TGF-β signaling, G6PD-deficient HAEC and SC monocytes were treated with TGF-β inhibitors. There was a significant trend of decreased monocyte-endothelial adhesion observed in TGF-β inhibitor groups compared to groups treated with G6PD inhibitors alone (Figure 3E). Thus, TGF-β signaling may be upstream of NOX components and regulate redox homeostasis in HAEC and prevent the adhesion of monocytes. 

### 2.6. NOX Inhibitors Impede Oxidative Stress and Monocyte-Endothelial Cell Adhesion in G6PD Deficient Cells 

**Figure 4:**
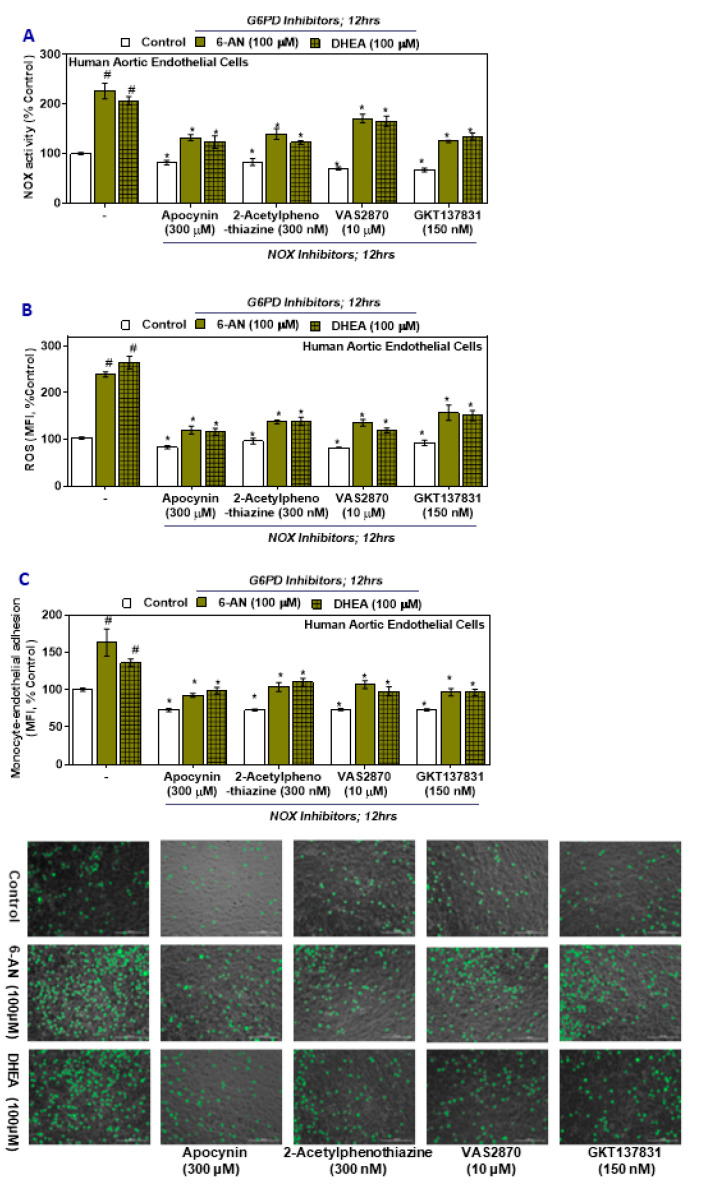
The effect of G6PD and NOX pharmacological inhibitors on NADPH oxidase activity, ROS in HAEC, and monocyte-endothelial cell adhesion. (**A**) NADPH oxidase activity. (**B**) ROS. (**C**) Phase-contrast images of HAEC and SC monocytic cells (scale bar: 200 μm). Results are mean ± SEM (*n* = 3–6). Significance at *p* < 0.05: #, compared with control–G6PD inhibitor groups; *, compared with control–NOX inhibitor groups.

Cells treated with either a G6PD inhibitor (6-AN or DHEA) followed by with treatment with NADPH oxidase inhibitors (apocynin, 2-acetylphenothiaznine, VAS2870, and GKT137831) showed significant upregulation of NOX activity, ROS in HAEC, and monocytic adhesion with the endothelium compared with the results observed in the groups treated with G6PD inhibitors alone (Figure 4A–C). However, while still being significantly higher (Figure 4A–C) compared to control HAEC and G6PD inhibitor treated control groups, the relative magnitude decreased following treatment with the NOX inhibitors. Inhibiting the NOX components, the primary source of superoxide, decreases oxidative stress and reduces monocytic adhesion with the HAEC. 

### 2.7. Effects of L-Cysteine Ethyl Ester (LC ee) on mRNA Expression of TGF-β/NOX, NOX Activity, ROS Levels, and Monocyte-Endothelial Adhesion

HAEC were pretreated with LC ee and then treated with H_2_O_2_. Cells treated with LC ee showed increased G6PD mRNA expression and activity and increased GSH content (Figure S2A–C). The expression of TGF-β1, TGF-β R1, TGF-β R2, NOX2, and NOX4 were all lower in cells treated with LC ee and H_2_O_2_ compared to those treated only with H_2_O_2_ (Figure S2A). LC ee treated cells also had lower NOX activity (Figure S2D), ROS levels (Figure S2E), and monocyte-endothelial cell adhesion (Figure S2F,G) when compared to control and groups treated with H_2_O_2_ alone. No change in cell viability was observed under any of our experimental conditions (data not shown). These results provide evidence that treatment with LC ee protects cells from oxidative damage by improving GSH levels, maintaining G6PD activity, and reducing monocyte-endothelial adhesion (Figure S2H).

## 3. Discussion

Glucose-6-phosphate dehydrogenase (G6PD) derived NADPH is thought to determine the rate of oxidative damage by recycling oxidized GSSG to GSH, a potent physiological antioxidant [15]. Various G6PD deficiency models have shown that it plays a principal role in altering redox homeostasis, impairing GSH, causing excess oxidative damage and defective vasodilation, and contributing to the progression of cardiovascular disease risk [2,8,12]. Recent studies have found that the presence of G6PD deficiency increases the risk of elevated systolic blood pressure and the incidence of CVD risk among populations in the USA, the Mediterranean area, and China [3,4,5]. In African Americans (AA), the G6PD A^−^ variant is recognized as predominantly associated with mild to a moderate enzyme deficiency (class III) with residual enzyme activity (10–60%) [4]. G6PD-deficient AA subjects showed elevated levels of OS and lower levels of GSH [9,16]. Furthermore, the incidence of G6PD deficiency among AA is 11–14%, compared with only 1% in Caucasians, and the AA population also has the highest incidence of CVD and 2–3 times the risk of stroke compared with other ethnic groups [7]. 

This study found that treatment with high glucose or palmitate decreases G6PD activity and increases levels of inflammatory cytokines (TNF and MCP-1) and cell adhesion molecules (ICAM-1 and VCAM-1) with concomitant increased adhesion of SC monocytes to HAEC. This provides a novel finding that a metabolic insult, such as hyperglycemia or exposure to free fatty acids, exacerbates inherited G6PD deficiency or status. It further exacerbates reduction in levels of GSH and increases in inflammatory (TNF and MCP1) and adhesion molecule expression and monocyte adhesivity in HAEC. This suggests that the presence of an inherited G6PD deficiency along with diabetes is a double burden and can further impair redox status and increase oxidative stress, assaults on the endothelium, and the risk of CVD.

This study also demonstrated increased TGF-β1 signaling and levels of NADPH oxidases in high glucose-treated HAEC with or without G6PD-deficient conditions. In addition, our studies also showed that inhibition of TGF-β signaling prevented the expression of NOX, ROS, and monocytic adhesivity to aortic cells. TGF-β/Smad signaling has been shown to increase ROS production and suppress antioxidant systems, including the synthesis of glutathione (GSH), an abundant intracellular free thiol, and several other antioxidant enzymes, thereby inducing oxidative stress or redox imbalance [17]. Studies have shown that ROS/RNS also upregulates TGF-β gene expression [18,19,20]. In particular, exogenous H_2_O_2_ has been shown to induce the expression of TGF-β in HUVEC [21]. Compared to healthy subjects, G6PD-deficient subjects have significantly higher TGF-β levels in cultured peripheral blood mononuclear cells [22]. This suggests that redox imbalance is an important contributor to TGF-β’s pathophysiologic effects and the induction of several NOX enzymes, mainly NOX4, in endothelial cells. 

**Figure 5:**
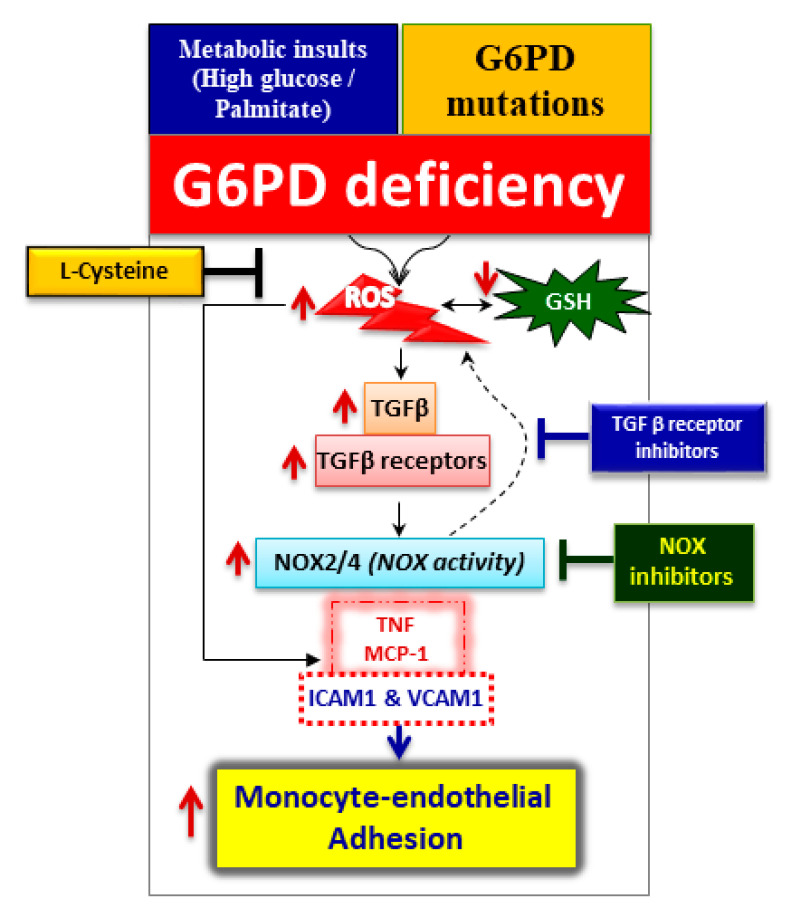
Schematic illustration of the proposed molecular mechanism of glucose-6-phosphate dehydrogenase (G6PD) deficiency in human aortic endothelium and monocytes. Metabolic insults (treatment with high glucose or palmitate) or G6PD gene mutations can cause G6PD deficiency. One possible mechanism is that G6PD deficiency decreases glutathione (GSH) and increases reactive oxygen species (ROS), which may activate transforming growth factor-beta1 (TGF-β1) signaling and NADPH oxidases (NOX). Activation of these factors generates excess oxidative stress, induces cytokines (TNF and MCP-1), upregulates cell adhesion molecules (ICAM-1 and VCAM-1), and favors monocyte-endothelial cell adhesion. Supplementation with L-cysteine (a GSH precursor) or ablation of the TGF-β signaling complex and NOX by inhibitors abolishes excess oxidative stress and inhibits monocyte-endothelial adhesion in G6PD-deficient cells. The key findings in our study demonstrate that G6PD deficiency plays an essential role in the initiation of a cardiovascular disease event via the TGF-β/NADPH oxidases/ROS signaling pathway.

G6PD deficiency induces NOX2/4 mRNA and activity in HAEC, whereas pharmacological inhibition of TGF-β receptor serine/threonine kinases (SB-505124, LY2157299, and LY2109761) reduces the expression of NOX activity and oxidative stress. In addition, blocking TGF-β signaling suppresses monocyte-endothelial adhesion, suggesting that TGF-β signaling may be upstream of NOX components and regulate redox homeostasis in HAEC (Figure 5). Previously it was shown that TGF-β/Smad signaling upregulates several NOX enzymes [17] and also G6PD deficiency activates monocytes and alters macrophage polarization [23]. Endothelial cells are capable of undergoing endothelial to mesenchymal transition (EndMT), a newly recognized type of cellular transdifferentiation. Transforming growth factor-1, which is considered the primary EndMT inducer, plays a role in the pathogenesis of various diseases, including malignant, vascular, inflammatory, atherosclerosis, pulmonary arterial hypertension, and fibrotic disorders [24]. Similarly, it was shown that G6PD deficiency has been associated with human fibrotic diseases [25,26,27]. Future studies are needed to elucidate the role of G6PD deficiency in EndMT and myocardial fibrosis.

NADPH oxidase inhibitors (apocynin, 2-acetylphenothiaznine (2-APT), VAS2870, and GKT137831), were able to suppress oxidative stress and impede monocyte-endothelial cell adhesion in G6PD-deficient cells. The most widely used NOX inhibitor in experimental conditions is apocynin, but it requires myeloperoxidase with H_2_O_2,_ which facilitates the dimerization of apocynin and can prevent assembly of an active NOX enzyme complex [28]. 2-APT is a selective cell-active inhibitor of NOX1 that blocks the generation of ROS [29]. VAS2870 appears to be a pan-NOX inhibitor, as it prevents assembly or conformational change to active NOX complexes, but it also has potential off-target effects [30]. GKT137831 has been shown to be a selective inhibitor of NOX1 and NOX4 isoforms [31]. The current challenge is that we lack isoform-specific NOX inhibitors. G6PD deficiency is associated with increased ROS production by NOX. Hence, it is suggested that the blockade of NOX-derived ROS using the NOX inhibitors may reduce the activation of endothelium and thus attenuate monocyte-endothelial adhesion (Figure 5). 

In this study, H_2_O_2_ induced TGF-β/NOX2, NOX4, and oxidative stress. The endothelium was partially protected from monocytic cell adhesion by pretreatment with L-cysteine ethyl ester (cell-permeable) (Figure S2). Hence, it is possible that G6PD deficiency-induced cellular dysfunctions could be prevented by supplementation with the micronutrient L-cysteine, which directly boosts the levels of the major antioxidant GSH and increases the transcription of G6PD [32,33,34,35], because G6PD deficiency increases oxidative stress and adversely affects the endothelium by activating TGF-β/Smad/NOX components. Future studies are needed to investigate whether supplementation with L-cysteine can be beneficial to the G6PD-deficient population.

Elevated oxidative stress, enhanced vascular inflammation, and immune cell infiltration contributes significantly to pro-atherogenic responses in diabetes [12]. In support of these observations, significant decreases in G6PD activity were observed in aortic endothelial cells and animal tissues due to hyperglycemia or diabetes [36,37]. In addition, the activation of TGF-β signaling and NADPH oxidases worsens the condition of diabetes. Meta-analysis and epidemiological studies have also shown that having a G6PD deficiency can increase the risk of developing diabetes and CVD [3,5]. In this study, we found that treatment with high glucose or palmitate per se induces a metabolic insult that causes G6PD deficiency along with oxidative stress (Figure S1). 

## 4. Materials and Methods 

### 4.1. Materials 

Human aortic endothelial cells (HAEC) (Lonza, CC-2535) (Lonza, Inc., Walkersville, MD, USA) were cultured using an Endothelial Growth Medium-2 BulletKit (Lonza) and grown to confluence at 37 °C in a humidified atmosphere containing 5% CO_2_. The culture was passaged according to standard procedures. For experiments, HAEC were used within 24 h after reaching confluence, between passages 4 and 6. Human monocyte/macrophage cells (SC) (American Type Culture Collection, Manassas, VA, USA; CRL-9855) were cultured in RPMI 1640 medium containing 10% (*v*/*v*) heat-inactivated fetal bovine serum, 100 unit/mL penicillin, 100 μg/mL streptomycin, 5.5 mM glucose, 12 mM sodium carbonate, 12 mM HEPES, and 2 mM glutamine. The culture was grown and maintained at 37 °C in a humidified atmosphere containing 5% CO_2_. Cells were counted using the Trypan Blue method before all treatments. The number of cells was maintained at around one million per milliliter of media. All chemicals, unless otherwise specified, were purchased from Sigma (St. Louis, MO, USA).

### 4.2. Cell Culture and Treatments

HAEC were treated with either high glucose (HG; 25 mM) or palmitate (200 µM) for 24 h in basal medium (without serum or any growth factors), respectively. Mannitol (19.5 mM) used as an osmolarity control for the HG group since the concentration of glucose used for the control group was 5.5 mM. Under uncontrolled diabetic conditions, blood glucose levels can become elevated to levels as high as 30 mM. Equimolar BSA was used as a control for the BSA conjugated palmitate group. 

HAEC and SC monocytes (G6PD-normal cells) were treated with the G6PD inhibitors 6-aminonicotinamide (6-AN; 100 μM) or dehydroepiandrosterone (DHEA; 100 μM) for 12 h in a basal medium; at 12 h, either NOX inhibitors (apocynin (300 μM), 2-acetylphenothiazine (300 nM), VAS2870 (10 μM), GKT137831 (150 nM)) or TGF-β receptor inhibitors (SB-505124 (150 nM), LY2157299 (100 nM), and LY2109761 (100 nM)) were added. All of the abovementioned pharmacological inhibitors were purchased from Cayman Chemicals (Ann Arbor, MI, USA). 

Cells were pretreated with L-cysteine ethyl ester (LC ee; 300 μM) for 12 h in a basal medium [38,39]; at 6 h, H_2_O_2_ (25 µM) was added to induce oxidative stress.

### 4.3. G6PD siRNA Knockdown Assay 

Endogenous G6PD was knocked down in HAEC and SC cells as follows: 100 nM siRNA G6PD (Santa Cruz Biotechnology, sc-60667) (Santa Cruz Biotechnology, Santa Cruz, CA, USA) and 6  µL Lipofectamine™ 2000 transfection reagent (Invitrogen, Carlsbad, CA, USA) were diluted in 100  μL transfection media (Santa Cruz Biotechnology) and incubated for 10  min. The diluted siRNA was combined with diluted lipofectamine, followed by an additional incubation at room temperature for 30 min to allow siRNA complexes to form before addition to the cells. Cells were incubated with siRNA medium for 4 h at 37 °C, followed by incubation with complete fresh media for the next 24  h. The double-stranded control siRNA-A (Santa Cruz Biotechnology, sc-37007) does not match any other current sequences used as a control in the experiments [34,35,40]. After the transfection procedure, G6PD-deficient cells were treated with basal medium at different time points, similar to the treatment protocol described elsewhere in this paper.

### 4.4. Cell Viability Assay

Cell viability was determined using the Alamar Blue reduction bioassay [41]. This method is based on Alamar Blue dye reduction by live cells. Briefly, cells were plated into 96-well plates after treatment following the above-described protocols, AlamarBlue^®^ Cell Viability Reagent (DAL1100, ThermoFisher Scientific, Waltham, MA, USA) was added, and the cells were incubated at 37 °C in the dark for 4 h. Absorbance was read at 590 nm using a plate reader. Data are expressed as a percentage of the viable cells counted compared to the total cells counted.

### 4.5. Monocyte-Endothelial Adhesion Assay

HAEC were plated and allowed to grow to confluent monolayers and then treated according to the protocol, as mentioned above. Monocytes (SC cells) were labeled with 8 μM CellTracker Green (CMFDA; Invitrogen, Eugene, OR, USA) and then treated following the protocol used for the HAEC. After treatment, 1 × 10^6^ monocytes were added to the endothelial monolayers and incubated at 37 °C for 45 min. The non-adherent cells were washed away with EC media and collected. Phase-contrast images of HAEC and monocytic cells were created using a Cytation 5 Cell Imaging Multi-Mode Reader with a 10× microscope objective and then merged. The merged images were used to quantify Celltracker Green labeled monocytes in the green channel. In each image, the total green signal associated with the level of monocyte adherence was quantified using Gen5 Image+ software (BioTek, Winooski, VT, USA). In addition, both adherent cells and non-adherent cells were lysed in 0.2% Triton X for quantification. The fluorescent intensity of the monocytes added to the monolayer (input), as well as that of the non-adherent cells, was measured at an excitation of 485 nm and an emission of 528 nm. Results are expressed as a percentage of control. 

### 4.6. Cellular Reactive Oxygen Species (ROS) Measurement

Cells in a black 96-well plate were incubated with chloromethyl-2′,7′-dichlorodihydrofluorescein diacetate (CM-H_2_DCFDA; 5 μM final concentration) in Hank’s balanced salt solution (HBSS) for 30 min at 37 °C in the dark. After being washed, the cells were stained with a nuclei dye, Hoechst 33342 (Molecular Probes, Eugene, OR, USA), for another 10 min at 37 °C. The cells were washed in HBSS, and the fluorescence was analyzed with at excitation/emission of 485/525 nm using a multi-detection microplate reader (Synergy HT, BioTek). The signal of CM-H_2_DCFDA was normalized to nuclei staining (excitation/emission at 350/470 nm). The change in intracellular ROS levels was plotted as mean fluorescence intensity (MFI).

### 4.7. Analysis of mRNA Expression Using Quantitative PCR

Total RNA extraction from HAEC was performed using TRIzol reagent (Invitrogen, Carlsbad, CA, USA). The concentration and quality of the extracted RNA were determined on a NanoDrop spectrophotometer (Thermo Scientific, Waltham, MA, USA). RNA (1 μg) from each sample was reverse transcribed according to the manufacturer’s instructions using a High Capacity RNA-To-cDNA kit (Applied Biosystems, Foster City, CA, USA) to synthesize cDNA. qPCR was performed using Applied Biosystems™ TaqMan™ [41]. Individual quantitative RT-PCR was performed with the gene-specific primer/probe sets, as shown in supplementary Table S1. The relative amount of mRNA was calculated using the relative quantification (ΔΔCT) method. The relative amount of each mRNA was normalized to the housekeeping gene *GAPDH*. In accordance with the requirements of the Minimum Information for Publication of Quantitative Real-Time PCR Experiments (MIQE) guidelines, technical replicates (*n* = 3) and biological replicates (*n* = 4) were included in all of our experiments. Data were analyzed using the comparative CT method, and the fold change was calculated using the 2^−ΔΔCT^ method with a 7900HT Real-Time PCR system and software (Applied Biosystems). The results are expressed as relative quantification (RQ).

### 4.8. Preparation of Whole-Cell Extracts 

For whole-cell extraction, after treatment the cells were washed twice with ice-cold PBS and lysed in RIPA buffer (50 mM Tris pH 8, 150 mM NaCl, 1% NP-40, 0.5% deoxycholic acid, and 0.1% SDS) supplemented with protease and phosphatase inhibitors (1 mM PMSF, 5 μg/mL leupeptin, 2 μg/mL aprotinin, 1 mM EDTA, 10 mM NaF, and 1 mM NaVO_4_). Lysates were then centrifuged for 10 min at 10,000× *g* at 4 °C. Supernatants were collected and the protein concentrations determined using a BCA assay kit (Pierce/Thermo Scientific, Rockford, IL, USA) for the various assays.

### 4.9. NADPH Oxidase Activity 

The NADPH oxidase activity of the cell lysates was determined following the method of Abid et al. [42]. Protein samples (50 μg) diluted in a reaction mixture containing 250 mM HEPES (pH 7.4), 120 mM NaCl, 5.9 mM KCl, 1.2 mM MgSO_4_ (7H_2_O), 1.75 mM CaCl_2_ (2H_2_O), 11 mM glucose, 0.5 mM EDTA, and 5 μM lucigenin were loaded onto a white 96-well plate. NADPH (100 μmol/L) was added to the cell lysates. Photon emission occurs from the chromogenic substrate lucigenin as a function of acceptance of an electron/O_2_^−^ generated by the NADPH oxidase complex; luminescence was measured every 60 s for 15 min using a Synergy HT microplate reader. Results are expressed as a percentage of control.

### 4.10. G6PD Activity

G6PD activity was measured in cellular extracts using a colorimetric-based assay (MAK015, Sigma; Glucose-6-Phosphate Dehydrogenase Activity Assay Kit), which is a simple, sensitive, and rapid assay that detects the activity of G6PDH in a variety of samples. In this kit, glucose-6-phosphate is oxidized to generate a product, which is specifically detected by a colorimetric assay measured at 450 nm. The G6PDH Activity Assay Kit can detect amounts of G6PDH as low as 0.04 milliunit per well. G6PDH activity is reported as nmol/min/mL (milliunit/mL). One unit is the amount of enzyme that catalyzes the conversion of 1.0 mmole of glucose-6-phosphate to 6-phosphoglucono-d-lactone and generates 1.0 mmole of NADH per minute at 37 °C. All appropriate positive controls and standards, as specified by the manufacturer’s kit, were used.

### 4.11. GSH Assay

GSH levels were quantified using a fluorimetric method (CS1020, Sigma; Glutathione assay kit, Fluorimetric). The cell extract for GSH was prepared according to the manufacturer’s protocol, the total protein was estimated using the BCA method, and then 50 μg of protein was used for assays [40,43]. The amount of reduced glutathione present in the sample is determined from the standard curve (linear regression analysis indicates the amount of fluorescence per nmol of the standard).

### 4.12. Western Blot Analysis

Equal amounts of proteins (20 μg) were separated on 10% SDS-PAGE and transferred to a polyvinyl difluoride (PVDF) membrane [41]. Membranes were blocked at room temperature for 2 h in a blocking buffer containing 1% BSA to prevent non-specific binding and then incubated with an appropriate primary antibody (supplementary Table S2). The membranes were washed in TBS-T (50 mmol/L Tris–HCl, pH 7.6, 150 mmol/L NaCl, 0.1% Tween 20) for 30 min and incubated with the appropriate HRP-conjugated secondary antibody (1:5000 dilution) for 2 h at room temperature. The protein bands were detected using ECL detection reagents (Thermo Scientific, Rockford, IL, USA) and exposed to blue X-ray film (Phenix Research Products, Candler, NC, USA). All of our immunoblot experiments included technical replicates (*n* = 2) and biological replicates (*n* = 4). Western blot scans were analyzed using ImageJ software (developed by Wayne Rasband, National Institutes of Health, Bethesda, MD, USA; access on 01/07/2020, available at http://rsb.info.nih.gov/ij/index.html). Densitometry analyses of Western blots were normalized to β-actin (ratio).

### 4.13. Statistical Analysis and Software

The data were subjected to one-way analysis of variance (ANOVA) followed by Tukey’s multiple comparisons test to assess the significance between control and experimental groups. The data are expressed as means ± standard error of the mean (SEM) and were considered statistically significant at *p* < 0.05. All analyses were performed using GraphPad Prism version 6.00 for Windows (GraphPad Software, La Jolla, CA, USA).

## 5. Conclusions

In conclusion, G6PD deficiency decreases GSH and increases ROS, which may activate TGF-β1 signaling and NADPH oxidases. Activation of these factors generates excess oxidative stress, induces cytokines (TNF and MCP-1), upregulates cell adhesion molecules (ICAM-1 and VCAM-1), and favors the adhesion of leukocytes to the endothelial monolayer (Figure 5). This cellular dysfunction is partially or completely preventable by supplementation with L-cysteine (a GSH precursor) or ablation of the TGF-β signaling complex and NOX by inhibitors abolishes excess oxidative stress and inhibits monocyte-endothelial adhesion in G6PD-deficient cells (Figure 5). The key findings in our study demonstrate that G6PD deficiency plays an essential role in the initiation of a cardiovascular disease event via the TGF-β/NADPH oxidases/ROS signaling pathway. Early intervention may reduce the risk of cardiovascular disease in the susceptible G6PD deficient population.

## Supplementary Materials

Supplementary materials can be found at https://www.mdpi.com/1422-0067/21/20/7474/s1. Supplementary Table S1. List of FAM-labeled TaqMan^®®^ primer/probe sets used for quantitative RT-PCR analysis. Supplementary Table S2. List of antibodies and dilutions used for Western blot analysis. Figure S1. (A–I) The effect of metabolic insults created by treatment with high glucose (HG; 25 mM) or palmitate (200 µM) on G6PD, TGF-β1, TGF-β1 receptors, and NADPH oxidases (NOX) in human aortic endothelial cells (HAEC) and monocyte-endothelial cell adhesion. Figure S2. (A–H) The effect of L-cysteine ethyl ester (LC ee) on TGF-β/NOX in H2O2-treated HAEC and monocyte-endothelial cell adhesion.

## Abbreviations

6-AN	6-aminonicotinamide
AA	African American
CAM	Cell adhesion molecule
CVD	Cardiovascular disease
DHEA	Dehydroepiandrosterone
ED	Endothelial dysfunction
G6PD	Glucose-6-phosphate dehydrogenase
GAPDH	glyceraldehyde-3-phosphate dehydrogenase
GSH	Glutathione
HAEC	Human aortic endothelial cells
HG	High glucose
ICAM-1	Intercellular adhesion molecule 1
LC ee	L-cysteine ethyl ester
MCP-1	monocyte chemoattractant protein 1
NO	Nitric oxide
NOX	NADPH oxidase
OS	Oxidative stress
RBC	Red blood cell
ROS	Reactive oxygen species
TGF-β	Transforming growth factor-beta
TGF-βR1	TGF-β receptor 1
TGF-βR2	TGF-β receptor 2
TNF	tumor necrosis factor
VCAM-1	Vascular adhesion molecule 1
