# Supplementary material for: Glucose-6-Phosphate Dehydrogenase Deficiency Activates Endothelial Cell and Leukocyte Adhesion Mediated via the TGFβ/NADPH Oxidases/ROS Signaling Pathway
Source: Int J Mol Sci. 2020 Oct 10;21(20):7474. doi: 10.3390/ijms21207474 (PMC7589139; doi:10.3390/ijms21207474)
Supplement: Supplementary file 1 [file ijms-21-07474-s001.pdf]

Supporting information

Glucose-6-phosphate dehydrogenase deficiency activates endothelial cell and leukocyte adhesion mediated via the TGFβ/NADPH oxidases/ROS signaling pathway.

Rajesh Parsanathan and Sushil K. Jain*

Department of Pediatrics and Center for Cardiovascular Diseases and Sciences, Louisiana State University Health Sciences Center-Shreveport, 1501 Kings Highway, Shreveport, Louisiana 71130, USA

Supplementary Table S1. List of FAM-labeled TaqMan® primer/probe sets used for quantitative RT-PCR analysis.

**Gene symbol**	**Gene Name**	**Assay ID**
G6PD	glucose-6-phosphate dehydrogenase	Hs00166169_m1
TGF-β1	Transforming growth factor-beta1	Hs00998133_m1
TGF-β1 R1	Transforming growth factor-beta1 receptor 1	Hs00610320_m1
TGF-β1 R2	Transforming growth factor-beta1 receptor 2	Hs00234253_m1
NOX2	NADPH oxidase 2	Hs00166163_m1
NOX4	NADPH oxidase 4	Hs01379108_m1
GAPDH	glyceraldehyde-3-phosphate dehydrogenase	Hs02786624_g1

Supplementary Table S2. List of antibodies and dilutions used for Western blot analysis.

**Antibody designation**	**Type**	**Antibody dilution and incubation time**	**Molecular weight (kDa)**	**Supplier (Catalog #)**
G6PD	Rabbit Polyclonal	1:1000, 4° C overnight	~ 60	Abcam (ab993)

TGF-β1	Mouse Monoclonal	1:400, 4° C overnight	~ 25	Santa Cruz (sc-52893)
TGF-β1 R1	Mouse Monoclonal	1:400, 4° C overnight	~ 53	Santa Cruz (sc-518018)
TGF-β1 R2	Mouse Monoclonal	1:400, 4° C overnight	~ 67	Santa Cruz (sc-17792)
NOX1	Mouse Monoclonal	1:400, 4° C overnight	~ 59	Santa Cruz (sc-518023)
NOX2 /gp91phox	Rabbit Polyclonal	1:1000, 4° C overnight	~ 60	Abcam (ab31092)
NOX4	Rabbit Monoclonal	1:1000, 4° C overnight	~ 63	Abcam (ab109225)
ICAM-1	Mouse Monoclonal	1:1000, 4° C overnight	~ 95	Abcam (ab2213)
VCAM-1	Mouse Monoclonal	1:400, 4° C overnight	~ 110	Santa Cruz (sc-20070)
MCP-1	Rabbit Polyclonal	1:1000, 4° C overnight	~ 11	Abcam (ab9779)
TNF-α	Mouse Monoclonal	1:1000, 4° C overnight	~ 25	Abcam (ab1793)
β-actin	Mouse Monoclonal - HRP	1:25000, 2 h RT	~ 42	Abcam (ab49900)
Goat anti-Rabbit-HRP Secondary Antibody		1:4000, 2 h RT	NA	Millipore (12-348)
Goat anti-Mouse-HRP Secondary Antibody		1:4000, 2 h RT	NA	Bio-Rad (170-6516)

Figure S1.

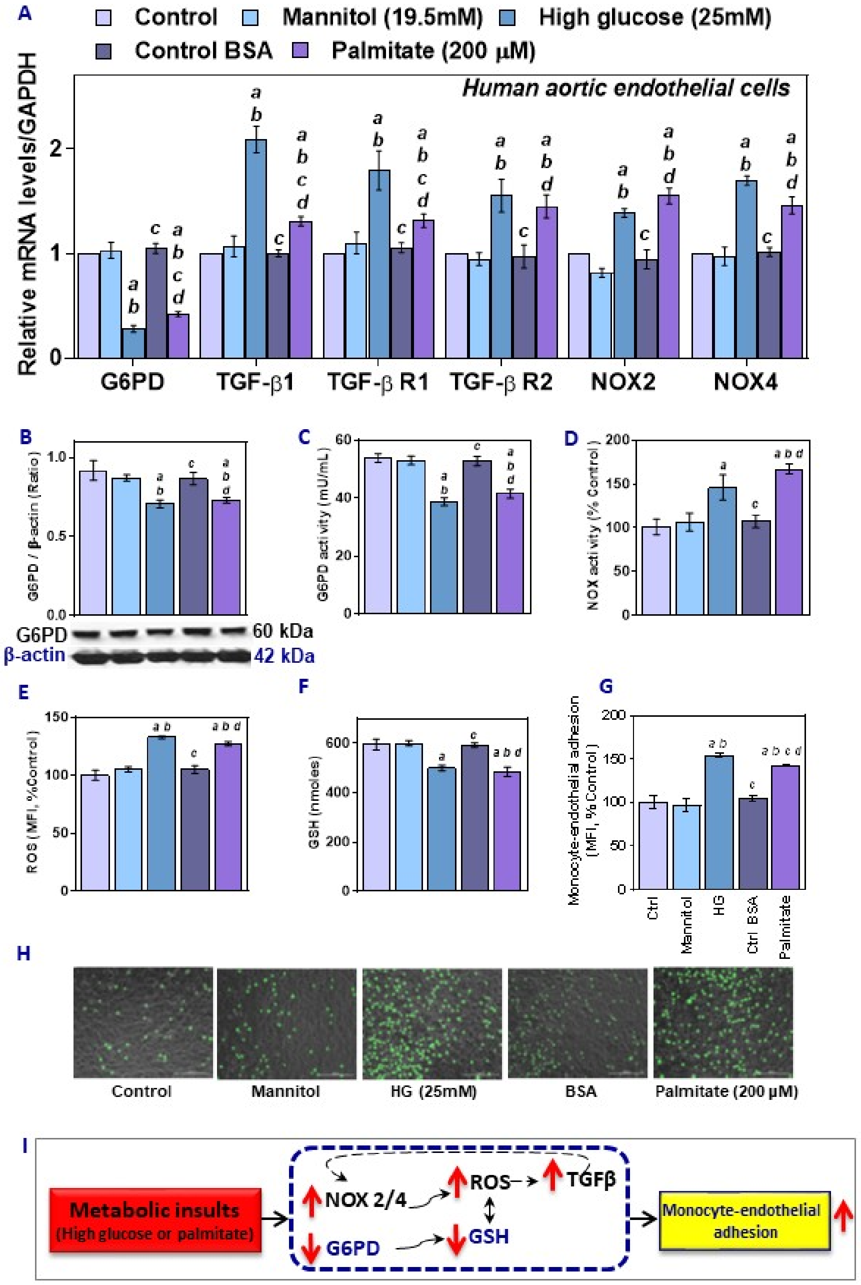

Figure S1. The effect of metabolic insults created by treatment with high glucose (HG; 25 mM) or palmitate (200 µM) on G6PD, TGF-β1, TGF-β1 receptors, and NADPH oxidases (NOX) in human aortic endothelial cells (HAEC) and monocyte-endothelial cell adhesion. A, RT-qPCR performed to assess the level of target genes as indicated. B and C, G6PD protein levels by Western blot and G6PD enzyme activity. D, NADPH oxidase activity. E, ROS and F, GSH content. G and H, Phase-contrast images of HAEC and SC monocytic cells (scale bar: 200 µm). I, Schematic showing that metabolic insults such as treatment with high glucose or palmitate mitigate G6PD, increase oxidative stress, and favor monocyte-endothelial cell adhesion. Results are mean ± SEM (n=3-6). Significance at $p<0.05$: a, compared with control; b, compared with mannitol; c, compared with high glucose; d, compared with control BSA.

Figure S2.

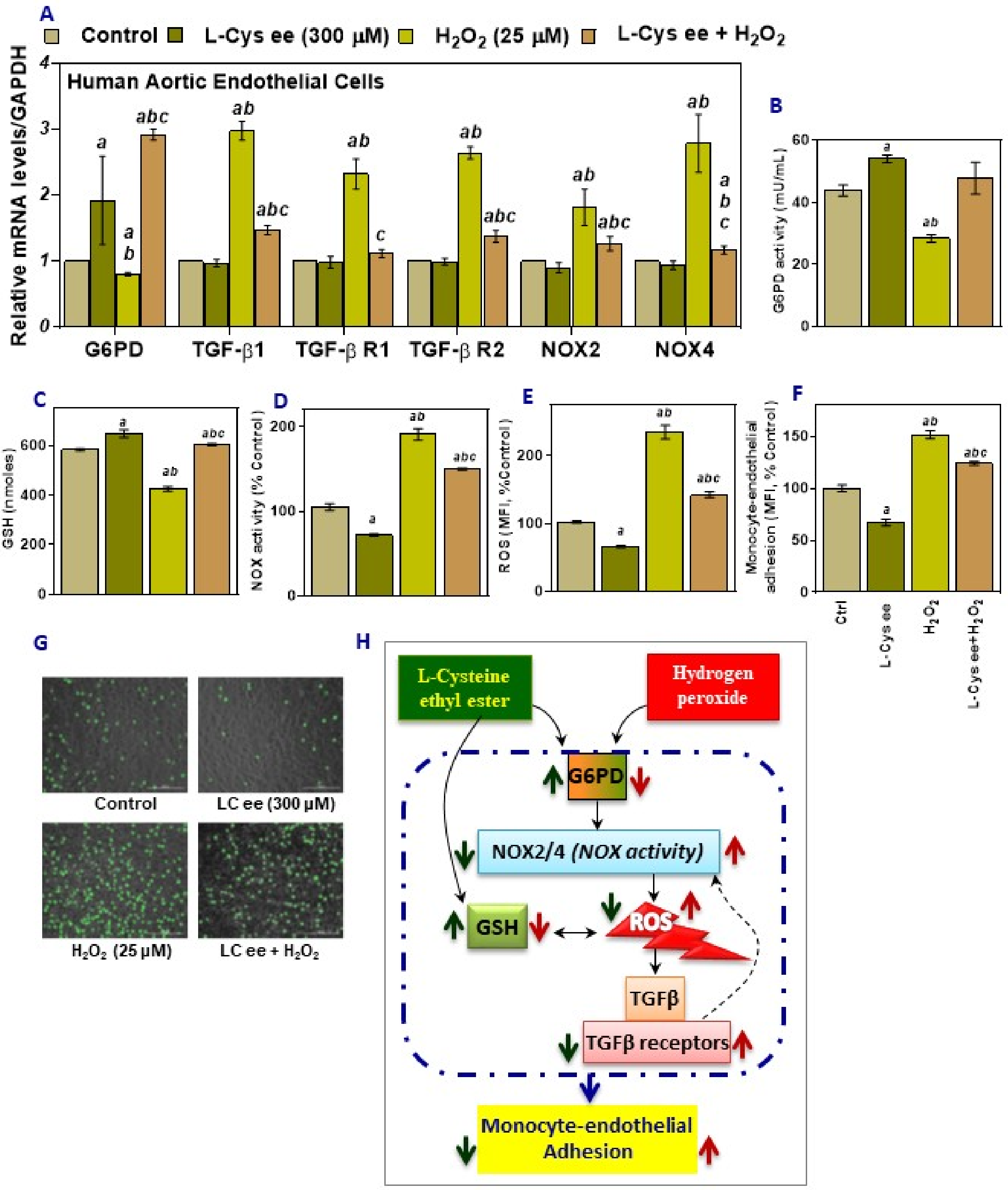

Figure S2. The effect of L-cysteine ethyl ester (LC ee) on TGF-β/NOX in H_2O_2-treated HAEC and monocyte-endothelial cell adhesion. A, RT-qPCR performed to assess the level of target genes as indicated. B, G6PD activity and C, NADPH oxidase activity. D, GSH content and E, ROS. F and G, Phase-contrast images of HAEC and SC monocytic cells (scale bar: 200 µm). H, Schematic showing the positive effect of supplementation with cell-permeable cysteine over peroxide-induced oxidative stress and monocyte-endothelial cell adhesion. Results are mean ± SEM (n=3-6). Significance at p<0.05: a, compared with control; b, compared with L-cysteine ethyl ester; c, compared with hydrogen peroxide.
